# Insights into Carbon-Based Aerogels Toward High-Performance Lithium–Sulfur Batteries: A Review of Strategies for Sulfur Incorporation Within Carbon Aerogel Frameworks

**DOI:** 10.3390/gels11070516

**Published:** 2025-07-02

**Authors:** Yue Gao, Dun Liu, Yi Zhao, Dongdi Yang, Lugang Zhang, Fei Sun, Xiaoxiao Wang

**Affiliations:** 1 Collage of Food Science and Technology, Wuhan Business University, Wuhan 430056, China; 2 State Key Laboratory of New Textile Materials and Advanced Processing Technologies, School of Textile Science and Engineering, Wuhan Textile University, Wuhan 430200, China; 3 Engineering Research Center of Technical Textile, Ministry of Education, College of Textiles, Donghua University, Shanghai 201620, China; 4 College of Textile Science and Engineering (International Institute of Silk), Zhejiang Sci-Tech University, Hangzhou 310018, China

**Keywords:** lithium-sulfur battery, carbon-based aerogels, polysulfides, shuttle effect, reaction kinetics

## Abstract

Lithium–sulfur batteries (LSBs), possessing excellent theoretical capacities, advanced theoretical energy densities, low cost, and nontoxicity, are one of the most promising energy storage battery systems. However, some issues, including poor conductivity of elemental S, the “shuttle effect” of high-order lithium polysulfides (LiPSs), and sluggish reaction kinetics, hinder the commercialization of LSBs. To solve these problems, various carbon-based aerogels with developed surface morphology, tunable pores, and electrical conductivity have been examined for immobilizing sulfur, mitigating its volume variation and enhancing its electrochemical kinetics. In this paper, an extensive generalization about the effective preparation methods of carbon-based aerogels comprising the combined method of carbonization with the gelation of precursors and drying processes (ambient pressure drying, freeze-drying, and supercritical drying) is proposed. And we summarize various carbon carbon-based aerogels, mainly including graphene aerogels (Gas) and carbon nanofiber (CNF) and carbon nanotube (CNT) aerogels as cathodes, separators, and interlayers in LSBs. In addition, the mechanism of action of carbon-based aerogels in LSBs is described. Finally, we conclude with an outlook section to provide some insights into the application of carbon-based aerogels in electrochemical energy storage devices. Based on the discussion and proposed recommendations, we provide more approaches on nanomaterials in high-performance liquid or state LSBs with high electrochemical performance in the future.

## 1. Introduction

Recently, due to the rapid development of electric vehicles and portable electronics, the demand for next-generation high-energy-density secondary batteries has increased dramatically [1,2,3]. Lithium–sulfur batteries (LSBs) possess energy densities as high as 2600 Wh kg^−1^ and a theoretical specific capacity of 1675 mAh g^−1^ and have received increasing attention as one of the most promising candidates [4,5]. However, the commercialization of LSBs is hampered by the severe “shuttle effect” of lithium polysulfides (LiPSs) and sluggish reaction kinetics [6,7]. For the purpose of conquering these issues, the mixing of sulfur with carbonaceous materials has become a mainstream of research after the pioneering work of Nazar’s group using mesoporous carbon (CMK-3)/S as cathodes [8,9]. Inspired by this, a series of nanocarbons have been investigated as sulfur hosts, functional layers of the separator, and interlayers to enhance the conductivity of the LSB system and to strengthen the physical inhibition for LiPSs [10,11]. The nanocarbons principally comprise carbon spheres, mesoporous carbon, graphene, carbon nanotubes (CNTs), carbon nanofibers, and many other carbon forms and structures [12,13,14]. For example, Zhou et al. [15] prepared a self-supporting fibrous graphene–sulfur (G-S) hybrid with good electrical conductivity as a cathode. And, the G-S blend with a highly porous network structure can provide highly conductive electron transport channels, rapid ion transport and short Li^+^ diffusion distances, and robust interaction with LiPSs. The blocking, adsorption, and reactivation of LiPSs can be achieved by introducing desirable functional groups to interact with LiPSs or by constructing composite lamellar separators in LSBs. In this regard, Zhai et al. [16] described a simple, scalable, and green process for porous graphene (PG)-modified separators. And LSBs assembled with a sulfur loading of 1.8–2.0 mg cm^−2^ and PG separators manifested a significantly high sulfur utilization of 86.5% at 0.05 C, an ultra-low self-discharge rate of 90% retention, and incremental rate capability.

Graphene is a two-dimensional material composed of a single layer of carbon atoms arranged in a honeycomb lattice structure. Its unique structure endows it with a series of world-leading properties, including high strength, high electrical conductivity, high thermal conductivity, and high specific surface area [17,18]. These properties make graphene highly promising for applications in electronic devices, energy storage, thermal management materials, environmental cleaning, catalysis, sensors, and other fields. Aggregation is a significant challenge facing graphene materials [19]. Due to its extremely high specific surface area and the van der Waals forces between its nanoplates, graphene is highly prone to aggregation, leading to severe performance degradation and significantly limiting its application potential [20,21]. To address the agglomeration issue, scientists have proposed methods such as surface modification to improve this situation, but the problem has not been fully resolved. Assembling 2 D graphene nanosheets into graphene aerogels (GAs) can effectively prevent graphene agglomeration and maximize the material’s superior mechanical, conductive, and high specific surface area properties [22,23].

Carbon aerogel is a synthetic, porous gel in which gas occupies 90–99% of the structure’s total volume. This highly porous, three-dimensional network does not shrink structurally. Its extremely high porosity confers it with unique properties, such as ultra-low density (0.003 g/cm^3^), low thermal conductivity, sound insulation, and a large specific surface area [24,25]. Compared to 2 D graphene, 3 D GAs have the features of unique structure, tunable pores, engineered densities, and the possibility of large-scale production [26,27,28]. Meanwhile, the GA framework retains an inherently prominent electron mobility and large specific surface area. Additionally, the hierarchical porous architecture of the GA can adsorb sufficient polysulfides and organic contaminants, and buffer the cells’ volume expansion during the charging and discharging process [29,30,31]. This makes GA an ideal candidate that is pertinent to use as electrocatalyst and functional separator for LSBs. Therefore, it is imperative to select appropriate functional nanomaterials (metal–organic frameworks, covalent organic frameworks, transition metal oxides, transition metal sulfides, and MXene, etc.) with heterostructures to immobilize on graphene bones to generate aerogels with 3 D network textures [32,33,34]. These modifications can catalyze the solid–liquid–solid conversion in S_8_-LiPSs-Li_2_S, thereby accelerating the reversible capacity and cycling performance of the cells [35,36,37]. For instance, Zhai et al. [38] ingeniously designed se vacancy-rich molybdenum selenide-modified GAs as both cathode host (MoSe_2−x_@GA/S) and freestanding interlayers (MoSe_2−x_@GA) for LSBs. The defect-rich MoSe_2−x_ can accelerate the nucleation and dissociation of Li_2_S, while the inserted bifunctional interlayer not only promoted the adsorption and transformation of LiPSs, but also regulated the homogeneous lithium deposition and inhibited the growth of lithium dendrites. And the LSBs assembled with the MoSe_2−x_@GA interlayer and electrode possessed a high initial discharge capacity of 1256.9 mA h g^−1^ at 0.2 C and an ultra-low decay ratio of 0.024% per cycle at 1.0 C over 1000 cycles. In addition to its use in cathodes and separators, GA is considered a promising host material for Li anodes due to its high electrical conductivity and large specific surface area.

Typically, 1 D and 2 D materials are introduced to assist in the construction of 3D frameworks, such as carbon nanofibers (CNFs), cellulose nanofibers, graphene oxide, etc., while 1D materials perform better than 2 D materials in terms of entanglement and gelation [39,40]. Among these materials, CNFs are commonly used as effective reinforcing agents for preparing novel Adv. Mater. (especially polymer-based materials) due to their excellent mechanical strength, high thermal conductivity, and self-lubricating properties [41]. This means that CNFs can be used to prepare nanocomposites with outstanding tribological properties through the reinforcement effect. Research has shown that using CNFs as a physical crosslinking agent enables atmospheric pressure drying and the preparation of ultra-lightweight yet robust aerogels [42,43].

**Figure 1:**
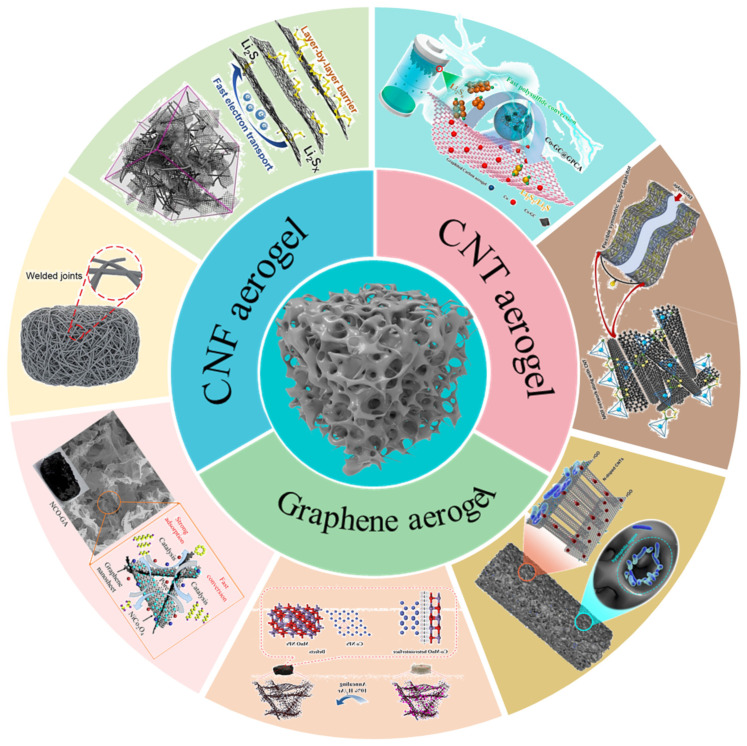
Schematic illustration of the carbon-based aerogels modifications used in LSBs.

There are numerous explorations reporting on the use of carbon-based aerogels in the functional separators, interlayers, and electrodes of LSBs [44,45,46]. The dominances of carbon-based aerogels in promoting the electrochemical performance of LSBs have been well documented [47,48,49]. However, to date, the review literature on the utilization of carbon-based aerogels in LSBs remains a gap. Based on the above, this review aims to systematically introduce the application of carbon-based aerogels in LSBs. Firstly, the effective methods to prepare carbon-based aerogels is discussed, including the combined method of carbonization with the gelation of precursors and drying processes (ambient pressure drying, freeze-drying, and supercritical drying). Subsequently, we outlined and analyzed the fabrication strategy and the use of carbon nanofiber and CNT aerogels as cathodes and interlayers for LSBs. Then, we highlighted recent evolutions on GAs in LSBs. In the extensive works on GAs in the as cathodes, functional separators, and interlayers of LSBs, we divided GAs into pure GAs, heteroatom-doped Gas, and composite GAs. Among them, the composite substances were introduced in detail from transition metal oxides (TMOs, like TiO_2_ and V_2_O_5_), transition metal sulfides (TMSs, such as MoS_2_, ZnS, and MoSe_2_), bimetallic compound, and multi-component, etc. Figure 1 broadly depicts the application of carbon-based aerogels for LSBs. Although carbon-based aerogels with various compositions and functions have been developed and successfully employed to lithium-ion batteries and LSBs, their further development in the field of electrochemical energy storage still faces challenges and future directions. Finally, we prospect the application of carbon-based aerogels in electrochemical energy storage devices.

## 2. Preparation of Carbon-Based Aerogels

### 2.1. Gelation of Precursors

**Figure 2:**
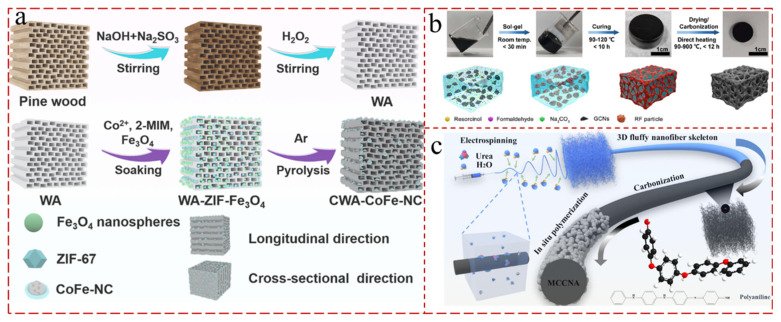
(**a**) Scheme showing the fabrication process of 3 D porous composite aerogel with decorated with CoFe-NC [64]. (**b**) A schematic diagram of the synthetic process used to prepare RF carbon aerogel monoliths by adding GCNs: the sol–gel, drying, and carbonization processes of the carbon aerogel and evolution of the RF clusters on GCNs. (A color version of this figure can be viewed online.) [60]. (**c**) Preparation of a multifunctional composite carbon nanofiber aerogel and its application in the monitoring and warning performance of human-related physiological signals [72].

Aerogels are typically fabricated via a sol–gel approach, a process comprising the conversion of molecular precursors into highly crosslinked inorganic or organic gels [50,51]. In general, there are three main steps in the synthesis of carbon-based aerogels: polymerization, drying, and carbonization [52,53,54]. In the polymerization step, the hydrogels are formed by polymerizing and cross-linking of the molecules. And, polymerization incorporates three separate chemical reactions [55,56]. Firstly, hydroxymethyl groups (-CH_2_OH) derived from aldehyde and hydroxyl groups are introduced via addition reactions. Subsequently, hydroxymethyl resorcinol leads to methylene (-CH_2_-) and methylene-ether (-CH_2_OCH_2_-) binds by the condensation reaction. Finally, the 3 D hydrogel structures are constructed by cross-linking and aggregation. In addition, various basic catalysts using sodium hydroxide (NaOH), sodium carbonate (Na_2_CO_3_), potassium carbonate (K_2_CO_3_), and calcium hydroxide (Ca(OH)_2_) are utilized in the initial addition phase [57,58,59]. Conventionally, organocarbon aerogels are produced by polymerizing formaldehyde and aromatic aldehydes (resorcinol, melamine, phenol, cresol, etc.) using various catalysts. For example, Gao et al. [60] synthesized carbon aerogel monoliths from resorcinol (R) and formaldehyde (F) using graphitic crystallite nanomaterials (GCNs) and Na_2_CO_3_ catalysts via the simple and ultrafast method (Figure 2b). In the gelation step, GCNs and Na_2_CO_3_ catalysts can significantly facilitate the addition reaction of RF, posing the gelation of RF within 30 min at room temperature. In the drying step, they also built transmission channels with dimensions up to hundreds, which allowed for the rapid and isotropic evaporation of water during heat drying hydrogels. Various functional nanoparticles such as polymers, inorganic salts, and ceramic nanoparticles were introduced into them during gel polymerization to synthesize carbon aerogels, which possessed outstanding pore size distribution, ordered pore structure, and excellent mechanical flexibility [61,62,63]. For example, Zeng et al. [64] elaborately constructed a 3 D porous carbon aerogel, with pine wood as a template, with highly dispersed CoFe alloy nanoparticles generated by the reaction of ZIF-67 and Fe_3_O_4_ nanorods in combination with hydrothermal and heat treatment processes (Figure 2a). The composite aerogel displayed a conductive network, which improved the conductivity loss, and sufficient interfaces to induce superior interfacial polarization.

Recently, graphene, CNTs, and carbon nanofibers have been proposed as precursors, which are easier to prepare than conventional polymers. There are various tactics for fabricating graphene aerogels, such as sol–gel, template, spacer-supported, self-supported, and substrate-based methods [65,66]. CNT-derived aerogels are promising candidates as conductive materials and are prepared by direct cross-linking via van der Waals interactions [67,68]. However, the directly obtained CNT aerogels exhibited serious intrinsic drawbacks, having a poor 3 D framework, unstable mechanical properties, and limited elasticity. The addition of surfactants and the introduction of polymer additives have been adopted to overcome these issues [69,70]. For instance, Chen et al. [71] manufactured reduced graphene oxide/CNT (RGO/CNTs) composite aerogels with high electrical conductivity and compressive strength by adding a CNT growth catalyst to RGO aerogels, followed by the direct growth of highly aligned CNT arrays between the RGO layers. The pillared CNT arrays between lamellar RGO layers provided the RGO/CNT aerogel with extremely high conductivity (214.7 S m^−1^) and compressive strength (73.6 kPa). Due to the abundant reserves, extremely high surface area, high porosity, and good mechanical properties, carbon nanofibers are another option for carbon-based aerogels. Yan et al. [72] employed electrostatic spinning, high-temperature calcination, and in situ polymerization to produce multifunctional carbon nanofiber composite aerogels with ultra-lightness, super elasticity, 3 D fluffiness, and interlayer porosity (Figure 2c).

### 2.2. Drying Processes

The drying process is an extremely critical stage in the preparation of carbon-based aerogels. The essence of drying gels is that the liquid ingredients of wet gels are superseded to attain a 3D solid network by gas (usually air) using appropriate drying techniques, which primarily involve atmospheric drying, supercritical drying, and freeze-drying methods [73,74]. Different drying methods have a great impact on the structural properties and performances of carbon-based aerogels.

#### 2.2.1. Ambient Pressure Drying

**Figure 3:**
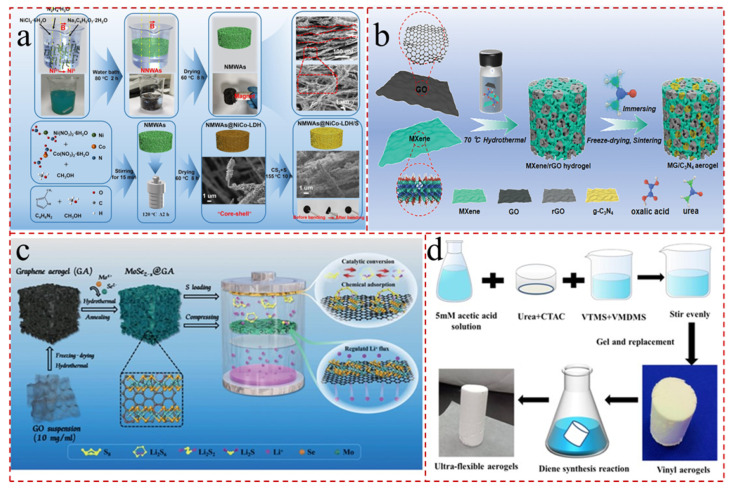
(**a**) Schematic illustration, SEM images, and digital photos for the synthesis of NMWAs, NMWAs@NiCo-LDH, and NMWAs@NiCo-LDH/S [28]. (**b**) Schematic diagram of synthesis of MG/C_3_N_4_ aerogel [96]. (**c**) The preparation and mechanism diagram of the MoSe_2−x_@GA/S interlayer integrated electrode [38]. (**d**) Preparation of ultraflexible aerogels by one-pot acid–base sol–gel synthesis [104].

Atmospheric pressure drying is a process that regulates surface tension by using low surface tension solvents. This process can generate superhydrophobic surfaces, thereby preventing excessive shrinkage and structural collapse during drying [75,76]. After capillary stress compression, the functional groups on adjacent surfaces will undergo further condensation. To solve the problem, the surface of the aerogel must be chemically treated to introduce some non-polar groups. For example, Wu et al. [28] initially synthesized ultra-light nickel microwire aerogels (NMWAs) and subsequently facilitated the growth of ultrathin porous nickel–cobalt-layered double hydroxide (NiCo-LDH) nanosheets on the surface of the aerogels (Figure 3a). Due the ordered porosity, abundant hydrophilic hydroxyl groups of NiCo-LDH, and the capillary action of NMWAs, the NMWAs@NiCo-LDH/S cathode displayed a distinguished rate performance (805.8 mAh/g at 5.0 C) and cycling stability (647.1 mAh g^−1^ over 700 cycles at 5.0 C with the capacity decay rate of 0.018% per cycle). However, atmospheric pressure drying easily destroys the network structure of aerogels during the drying process. This is due to the surface tension of liquids, the destruction of capillary forces between pores during gel drying, and the resulting internal stress differences. These factors can cause aerogels to collapse [77,78]. During the drying process of the gel, the hydroxyl groups on the surface of the skeleton particles undergo irreversible condensation, leading to shrinkage. Although this problem can be addressed by enhancing the strength of the skeleton and modifying the surface of the gel network, the effectiveness of these methods is not ideal.

However, atmospheric pressure drying can cause damage to the aerogel network structure during the drying process. This is due to the surface tension of the liquid, the destruction of capillary forces between pores during gel drying, and the resulting internal stress differences. These factors lead to structural collapse [79,80]. Conversely, supercritical drying reduces the surface tension to zero, keeps the aerogels from shrinking and collapsing, and preserves the intact skeletal structures by converting the drying medium into a supercritical state at a high temperature and pressure [81,82]. By removing the gas–liquid phase difference, the freeze-drying technique lowers the capillary force, preserving the dried sample’s volume and structure. Another crucial stage in controlling the material’s nanoscale shape is the freezing process. By reducing the impact of the gas–liquid phase, the freeze-drying process ensures that the dried aerogels maintain their volume and structure [83,84]. Another crucial stage in controlling the material’s nanoscale shape is the freezing process. Additionally, supercritical drying involves bringing the drying medium to a supercritical state at high temperatures and pressures. This process can successfully eliminate surface tension, stop the aerogels from shrinking and disintegrating, and preserve the intact skeleton structures. Therefore, it is recommended to prepare the necessary aerogels using the freeze-drying and supercritical drying procedures.

#### 2.2.2. Freeze-Drying

Due to the simple operation, time-saving feature and adjustable pore structure, the freeze-drying is quite widely used to manufacture carbon composite aerogels. For the preparation of three-dimensional porous materials, freeze-drying works well. This is due to the possibility of uniform distribution of the initial components in the initial suspension or solution. Therefore, the freezing process usually does not cause stratification or separation, producing a composite material with a homogeneous phase distribution [85,86,87]. The pore structure of aerogels plays an important role in inhibiting the shuttle behavior of LiPSs. Aerogels with a higher porosity and smaller pore sizes generally have better mechanical and dimensional stability, but their flux may be negatively impacted by the minuscule apertures [88,89,90].

Therefore, the electrochemical characteristics of LSBs are intimately linked to the right pore size. In this method, the solvent in the hydrogel is frozen to ice crystals, and the obtained ice crystals are subsequently sublimated and removed to form 3 D porous frameworks in the low-pressure environment [91,92]. Furthermore, differential segregation is typically not observed during the freezing process, and the phase distribution is homogeneous in the obtained composite aerogels [93]. This method can reduce the capillary forces by eliminating the discrepancies in the phase of the gas and liquid, engineer the micro-morphology of the aerogel, and reconcile the pore structures by controlling the direction of ice crystal growth and the rate of diffusion [94,95]. Yang et al. [96] prepared MXene/reduced graphene oxide/C_3_N_4_ (MG/C_3_N_4_) aerogels with a 3 D architecture by the low-temperature hydrothermal method, followed by freeze-drying and heat treatment, as sulfur carriers for the free-standing cathode of LSBs (Figure 3b). But the rapidly frozen samples contained small ice crystals, which could generate small pores and large pore surface area. The 3 D aerogel structure provides sufficient space for loading substantial quantities of active sulfur, thereby mitigating the associated volume changes. Additionally, it offers an adequate number of adsorption sites, facilitating charge transfer and ion diffusion.

The low concentration of precursors has the effect of promoting the growth of ice crystals, which in turn affects the porous structure of the aerogels [97,98]. Nevertheless, there are still some issues associated with the freeze-drying process. For instance, the slow rate of sublimation results in the necessity of multiple freezes to achieve optimal results. Meanwhile, Se vacancy-rich molybdenum selenide-modified graphene aerogels were created by Zhai et al. [38]. And the composite aerogels function as both freestanding interlayers (MoSe_2−x_@GA) and cathode hosts (MoSe_2−x_@GA/S) for LSBs (Figure 3c). Furthermore, the defect-rich MoSe_2−x_ can speed up the nucleation and dissociation of Li_2_S because of its sulfiphilic–lithiophilic characteristics. And the insertion of the bifunctional interlayer can both speed up the adsorption and conversion of polysulfides and control uniform lithium deposition, which limits the growth of lithium dendrites.

Nevertheless, in the freeze-drying method, the growth of solvent crystals, especially in the case of water as a solvent, can lead to the volume expansion of the crystals and increase the stresses in the gels [99,100]. Instead, these stresses are conducted inward from the surface layers of the crystals, causing the crystals to shrink and break up into small particles.

#### 2.2.3. Supercritical Drying

The supercritical state is defined as a liquid above a thermodynamic critical point. Due to the low critical temperature and pressure of carbon dioxide (CO_2_) and low-cost, non-toxic, and convenient reaction process, CO_2_ with low viscosity was employed for drying, which can allow for rapid drainage from the 3 D gel frame in the supercritical state [101,102,103]. During the drying process, the solvent is converted from its liquid phase to a supercritical fluid and is expelled from the gel, arising the construction of a porous 3 D gel framework. Du et al. [104] ingeniously synthesized ultraflexible and super hydrophobic silica aerogels through the one-pot acid–base sol–gel route and supercritical drying approach (Figure 3d). The acquired aerogels can be recovered completely without structure destruction over 20 cycles of compression–decompression with 90% strain. Compared with other drying techniques, supercritically dried aerogels have more affluent pores, uniform structures, and lager specific surface areas, which plays an important role in improving the electrochemical performance of electrodes. Lv et al. [105] prepared MXene/CNT/CNF aerogels by supercritical drying with a larger specific surface area compared to freeze-drying and atmospheric-pressure drying, which can bring about excellent electrochemical stability and high specific energy density.

### 2.3. Carbonization

**Figure 4:**
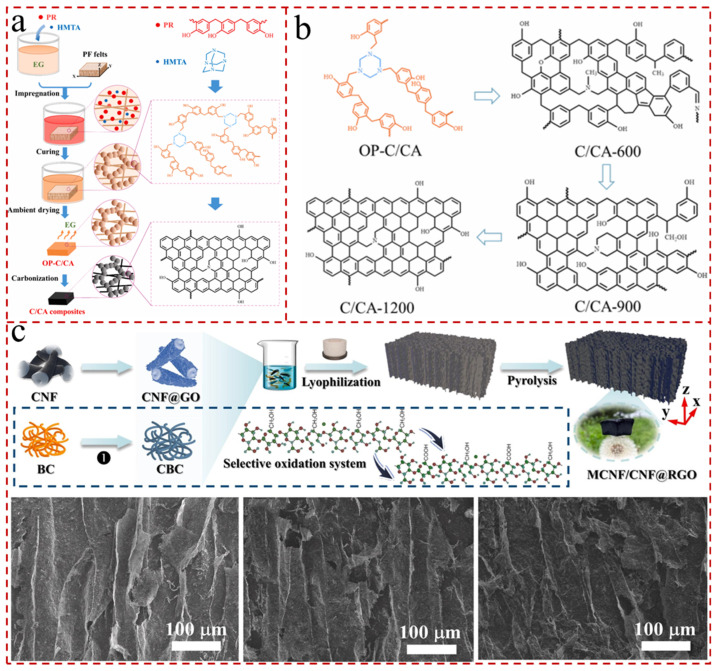
(**a**) Schematic of the preparation of the C/CA composites [108]. (**b**) Proposed molecular structure evolution of C/CA at different carbonization temperatures [108]. (**c**) Fabrication scheme of pure carbon aerogel: z refers to the oriented direction, and low magnification SEM images of CC600, CC700, and CC800 [113].

In addition to the previously mentioned direct use of carbon materials to obtain carbon aerogels, carbon aerogels are acquired by high-temperature post-treatment of the inert atmosphere (e.g., N_2_ or Ar). The pore structure of the aerogel is affected by the carbonization temperature and the heating rate, while, as the carbonization temperature increases from 873 to 1473 K, the dimensions of the aerogels can slightly vary and the volume of the micropores marginally decreases [106,107]. Ma et al. [108] prepared carbon fiber-reinforced carbon aerogel-like matrix (C/CA) composites at different carbonization temperatures from 600 to 1200 °C. The removal of residual organ functional groups from the C/CA precursor dominated at 600–750 °C carbonation, with a 17–22% difference in volumetric shrinkage compared to 1200 °C (Figure 4a,b). The mesopores dominate in the carbon aerogel at a low carbonization temperature (873 K), while the micropores govern in the carbon aerogel at a high carbonization temperature (1473 K), and the specific surface area of the carbon aerogel decreases significantly [109,110]. And, when the carbonization temperature exceeds 2273 K, the aerogel is completely graphitized [111]. In addition, the porosity and specific surface area of aerogels can be augmented using the implementation of physical or chemical activation techniques by potassium hydroxide (KOH), sodium hydroxide (NaOH), CO_2_, and water (H_2_O) [112]. The activation of activated carbon aerogels can control the surface area, pore volume, and pore size distribution of the products. And, common carbon-based aerogel materials include CNT aerogels, carbon nanofiber aerogels, multi-hollow carbon aerogels, and graphene aerogels. Su et al. [113] constructed composite aerogels containing carbon nanofibers@reduced oxide graphene (CNF@RGO), with the carbon nanofibers being derived from bacterial cellulose (BC) by heat treatment at various specific temperatures (600, 700, and 800 °C) (Figure 4c). The aerogels featured an ultra-light density of 3.6 mg/cm^3^ and showed the greatest performance at the carbonization temperature of 700 °C.

## 3. Carbon Nanofiber Aerogels

### 3.1. Synthesis of CNF Aerogel

Carbon nanofibers (CNFs) are 1 D carbon materials, mainly obtained by electrostatic spinning, which is one of the most established and widely adopted techniques among all the current strategies available for synthesizing one-dimensional nanofibers [114,115]. CNFs possess notable features of extremely high surface area and high porosity, which makes them robust and attractive candidates for many advanced electrodes in various batteries. A variety of polymers, including polypropylene cyanide (PAN) and polyvinyl alcohol (PVA), have been employed as precursors of CNFs [116,117]. Various CNFs with unique structures, comprising hollow, core–shell, multi-channels, island, and so on, can be prepared by adjusting the polymer viscosity, the process parameters, and specially the structured electrostatic spinning heads as well as the post-processing techniques [118,119].

There are two main synthetic methods for the preparation of carbon nanofiber aerogels, which are the electrostatic spinning–sol–gel–carbonation method and the sol–gel–carbonation method. The former converts synthetic polymers into nanofibers by the electrospinning method, followed by the sol–gel and carbonization processes. The latter directly transforms the natural polymer fibers into the sol–gel state and uses high temperatures to form carbon nanofiber aerogels [120,121]. Compared to synthetic polymers, biomass nanofiber aerogels show better environmental friendliness and easier operation [122].

### 3.2. Modified CNF Aerogel

**Figure 5:**
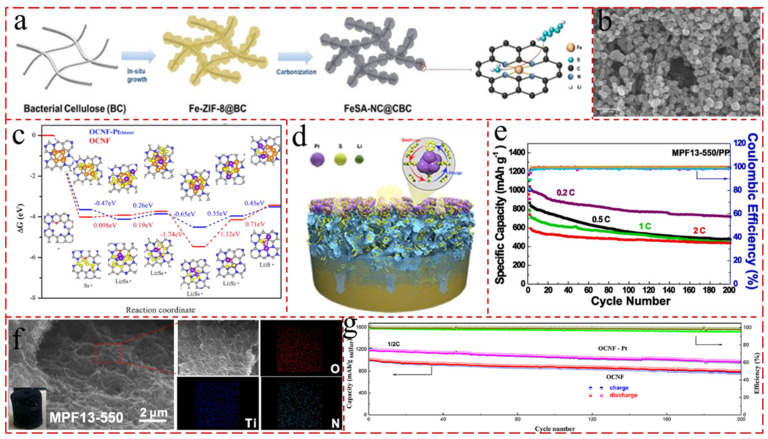
(**a**) Schematic illustration of the synthesis process of FeSA-NC@CBC [127]. (**b**) SEM images of FeSA-NC@CBC [127]. (**c**) Gibbs energy profiles of Li/S conversion chemistry [50]. (**d**) Schematic diagram of the mechanism of Pt nanoparticle-interfaced sulfur cathode in the redox process [50]. (**e**) Cycling performance of LSBs with MPF13-550/PP separators at the rates of 0.2, 0.5, 1.0, and 2.0 C [139]. (**f**) SEM and the corresponding selected area elemental mapping images of MPF13-550 and the digital image (inset) [139]. (**g**) Cycling performances of OCNF and Pt nanoparticle-interfaced Li/S cell [50].

Due to the flexible structure and highly conductive network, CNF aerogels are considered as an efficient sulfur host, which can accommodate the volume expansion of LSBs during cycling and to immobilize and physically capture the LiPSs [123,124]. Biomass is undoubtedly the most abundant, inexhaustible, and greenest energy source on the planet, which has attracted significant attention in the energy storage field. The biomass material fiber has attracted much attention due to its abundant reserves, renewability, low cost, lightweight, non-toxicity, and its inherent heteroatom doping [125,126]. Inspired by this, Lin et al. [127] synthesized Fe single-atom catalyst-functionalized N-doped carbon nanofiber (FeSA-NC@CBC) aerogels by using iron-doped 2-Methylimidazole zinc salt (ZIF-8) introduced in biomass BC as the precursors (Figure 5a,b). The obtained FeSA-NC@CBC aerogels were employed as the freestanding sulfur cathodes and can facilitate the conversion of S_8_-LiPSs-Li_2_S to address the LiPS shuttling problem because of the catalytic functions of FeSA-NC. Benefiting from the synergistic effects of highly active Fe-SAC to effectively chemisorb LiPSs and expedite the redox conversion kinetics, the LSBs assembled delivered a superior rate capability of 840 mAh g^−1^ at 2 C and a low-capacity decay rate of 0.042% per cycle after 500 cycles at 1 C. Cotton, a naturally occurring fiber, is the best choice for textiles both for military and civilian purposes due to its affordability, durability, and comfort [128,129]. By virtue of its low price, environmental friendliness, and rich atomic structure, waste recycled cotton is important in the carbon fiber process. Various elements such as oxygen, sulfur, and nitrogen, inherent in natural cotton fibers, can be retained in situ doped into carbon fibers during pyrolysis, which facilitates their effective capture and adsorption of LiPSs [130,131]. Ji et al. [50] utilized cotton as precursor and ingeniously designed an O and N-tailored carbon fiber aerogel (OCNF) with Pt as cathodes in LSBs (Figure 5c,d,g). The Pt nanoparticles were uniformly sprayed onto the S surface to form the electrocatalytic interface (Pt/S/OCNF) to create ion channels and facilitate the effective penetration of the electrolyte into the cathode. The Pt/S/OCNF cathode has a high sulfur utilization rate of 77.5%, an excellent rate capacity of 813.2 mAh g^−1^ (2.0 C), and a prominent long-cycling performance, with a capacitance retention of 82.6% and a decay of 0.086% per cycle after 200 cycles at 0.5 C. During the reduction of Li_2_S_4_ to Li_2_S_2_, the maximum endothermic Gibbs free energies were 1.32 eV (OCNF) and 0.55 eV (OCNF-Ptcluster), respectively, indicating the rate-limiting step during the discharge process. Additionally, the second plateau discharge process from Li_2_S_4_ to Li_2_S_2_ exhibits significant endothermicity, with the Gibbs free energies for OCNF-Ptcluster (0.55 eV and 0.45 eV) being significantly lower than those for OCNF (1.32 eV and 0.71 eV), indicating lower energy barriers for achieving rapid redox and chemical conversion kinetics.

As another biomass nanofiber, cellulose is the most widely available renewable resource from a variety of living organisms, including plants (trees), microorganisms (algae, fungi, bacteria), and animals (tunicates), etc. [132,133]. TEMPO-oxidated cellulose nanofibers (TOCNFs) obtained by TEMPO-mediated oxidation reactions have attracted great interest. Under the TEMPO oxidation reaction, the C_6_-hydroxyl group on the cellulose surface is site-selectively oxidized to C_6_-carboxyl groups. The obtained TOCNFs have good size (3–4 nm), excellent crystallinity (65–95%), high aspect ratio (>50), burgeoning modulus of elasticity (6–7 GPa), and intriguing tensile strength (200–300 MPa) [134,135]. Hu et al. [45] ingeniously designed a staphylo-Ni_3_S_2_ in situ embedded on a N-doped carbon nanofiber aerogel, TOCNFs as precursors, with a hierarchical pore structure as a sulfur host for LSBs. And, the polar Ni_3_S_2_ and N-doped carbon structure can facilitate the catalytic conversion of LiPSs and coordinate the 3 D nucleation of Li_2_S, which could diminish the reaction energy barrier. Therefore, the obtained cathode can maintain a high initial capacity (1080.2 mAh g^−1^) and excellent stability at 0.1 C. Zhang et al. [136] successfully prepared a cellulose-based high-pyrrole nitrogen-doped carbon aerogel (PNCA) using a simple and environmentally friendly in situ dissolution method. The resulting aerogel has a honeycomb-like three-dimensional structure and a high pyrrole nitrogen-doped configuration. DFT results indicate that the absolute adsorption energy of pyrrole nitrogen configuration pyrrole-like NG is the highest, indicating that pyrrole nitrogen has the strongest stability for different polysulfides and the strongest fixed adsorption capacity, which can greatly inhibit the process shuttle effect of polysulfides.

The characteristics of the electrospinning polymer solution, such as surface tension, electrical conductivity, volatility, and solvent viscosity, etc., can collectively influence the properties of the fibers [137,138]. These factors highlight that, by varying fluid properties, it is possible to produce various types of fibers as required. Lin et al. [139] synthesized a crumpled nitrogen-doped porous MXene–pyrrole–formaldehyde (MPF) composite aerogel through the freeze-drying and high-temperature (550 °C) carbonized methods, in which the monomers of formaldehyde and pyrrole induced by MXene formed polymer gels as precursors (Figure 5e,f). And the as-prepared MPF composite aerogel (MPF13-550), prepared by regulating the content of MXene and pyrrole–formaldehyde at 1:3, possessed a large specific surface area (179 m2 g^−1^) and high nitrogen doping (10.22 at.%). The LSBs assembled with the MPF13-550/PP separator manifested an excellent initial discharge capacity (1235 mA h g^−1^ at 0.1 C), fantastic rate capabilities (593 mA h g^−1^ at 2.0 C), and superior cycling performance (721 mA h g^−1^ at 0.2 C after 200 cycles).

CNF aerogels show unique structural features and favorable performance, but there are relatively few explorations for cathodes and separators in LSBs, which is mainly attributed to the complex and time-consuming operations. Compared to carbon nanofiber aerogels, CNT and graphene-based aerogels shine as cathodes, separators, and interlayers in LSBs.

## 4. Carbon Nanotube Aerogels

### 4.1. Synthesis and Characterization of CNT Aerogels

CNTs can be classified into single-walled CNTs (SWCNT) and multi-walled CNTs (MWCNT) according to their layers. There are many well-established methods to prepare CNT-based aerogels, among which the most widely used is the sol–gel assembly method. This method was published and proposed by Kim et al. [140] in 2013, involving the uniform dispersion of single-walled CNTs with sodium dodecylbenzenesulphonate as a surfactant, followed by freeze-drying. In this method, the dimensions and pore size of the aerogels can be tuned. Zou et al. [141] used a similar approach with poly(3-(trimethoxysilyl) propyl methacrylate) (PTMSPMA) to disperse and functionalize MWCNT, where the subsequent hydrolytic condensation of PTMSPMA can generate the formation of high-strength chemical bonds in MWCNTs.

CNT aerogels can possess a complete conductive network and extremely high specific surface area, which can improve sulfur loading and the utilization of active materials. And CNT aerogels have rich porous structures, which can accommodate the volume expansion of cathodes during cycling [142,143,144,145,146]. Notably, organic solvents can easily penetrate and be stored in the interior of CNT aerogels, which is conducive to suppress the shuttle effect of LiPSs through the physical and chemical interaction [147,148,149]. However, pure CNT aerogels have some drawbacks, including weak interactions with LiPSs and poor mechanical properties, which largely limit their extension to LSBs. Therefore, magnanimous functional materials are introduced into CNT aerogels to form composite aerogels to further improve and enhance electrochemical properties.

### 4.2. CNT Composite Aerogels for Cathodes

**Figure 6:**
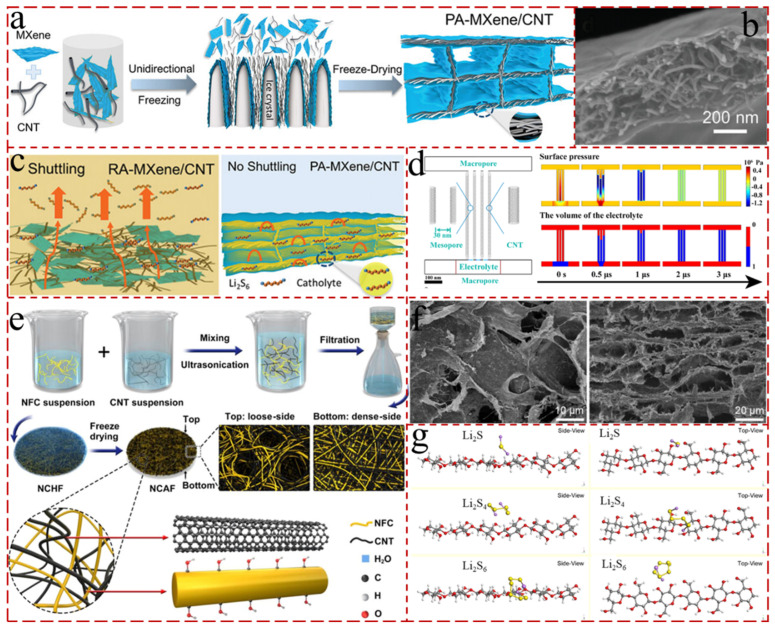
(**a**) The schematic of the assembly process of the PA-MXene/CNT aerogel by unidirectional freeze-drying [151]. (**b**) SEM images of the PA-MXene/CNT-50 aerogel [151]. (**c**) The schematic of LSBs with PA-MXene/CNT or RA-MXene/CNT as hosts and Li_2_S_6_ solution as catholyte. LiPS shuttling occurred in the RA-MXene/CNT host and was effectively suppressed by PA-MXene/CNT [151]. (**d**) Multiphysics simulation of the electrolyte absorption by the CNT aerogel [143]. (**e**) Schematic diagram illustrating the fabrication process of NCHF and NCAF [157]. (**f**) SEM images of the top layer and vertical section of NCAF-6/4 [157]. (**g**) Optimal configurations (side and top view) of Li_2_S, Li_2_S_4_, and Li_2_S_6_ molecules adsorbed on cellulose surfaces [157].

CNTs are intertwined with each other to form porous and isotropic structures with a high surface area (1291 m^2^ g^−1^) and excellent pore size distribution [150,151]. To the overcome inherent flaws of low strength and poor structural stability, the CNT aerogels are strengthened by incorporating specific polymers (e.g., PVA, chitosan, polypyrrole, and polyimide, etc.) or binders (polydimethylsiloxane (PDMS), ethylenediamine) into the sol [152,153,154]. In addition to adding binders, grafting CNTs with functional groups and creating chemical bonds between CNTs represent a powerful way to improve mechanical properties [142,155,156]. The rich pore sizes of CNT aerogels can accommodate sulfur and buffer volume expansion during the charge and discharge process, and their convenient channels can provide fast transport paths for electrons in LSBs. Zhang et al. [151] prepared a lamellar aerogel composed of MXene/CNT sandwiches with the unique parallel-aligned structures by unidirectional freeze-drying to enhance the cycling stability of high sulfur-loading batteries (Figure 6a). The multiple physical barriers formed by the layered structure, combined with the chemical trapping and catalytic activity of MXene, were able to effectively inhibit the shuttling of LiPSs under high sulfur loading, and more importantly, the micro- and mesopores, as 3 D hosts, can substantially confine the LiPSs. The assembled LSB delivered a high capacity of 712 mAh g^−1^ with a sulfur loading of 7 mg cm^−2^, and a superior cycling stability with 0.025% capacity decay per cycle over 800 cycles at 0.5 C (Figure 6b,c). Furthermore, Bai et al. [157] also finely engineered asymmetric nanofibrillated cellulose/CNTs (NFC/CNTs)-entangled aerogel films as cathodes in LSBs via vacuum filtration and lyophilization, which were integrated with high-aspect-ratio NFC and low-cost commercial CNTs (Figure 6e). The NFC/CNTs aerogels possess superior conductivity, intriguing mechanical strength, sufficient reactive hydroxyl groups, and asymmetric structures, which comprise loose and dense layers (Figure 6f). The loose structure had many large-sized pores that facilitated sulfur storage and electrolyte wetting, infiltration, and diffusion, while the dense architecture displayed a uniform distribution of small pores, which accelerated the inhibition of LiPSs and hindered their diffusion into the anode (Figure 6g). In addition to sulfur as an active material in LSBs, various liquid LiPSs has also been explored as active substances of LSBs to increase the active materials’ loading. For example, Fang et al. [143] proposed to drop a Li_2_S_8_ solution into CNTs and to fabricate a porous CNT aerogel@Li_2_S_8_ as cathode by the freeze-drying method (Figure 6d). The CNT aerogel had a rich mesoporous structure, which largely improved the utilization of the active material and enabled ultra-high active substance loading due to the extremely strong capillary effect. The CNT aerogel@Li_2_S_8_ cathode exhibited an ultrahigh areal-specific capacity of 22.9 mAh cm^−2^.

### 4.3. CNT Composite Aerogel for Interlayer

**Figure 7:**
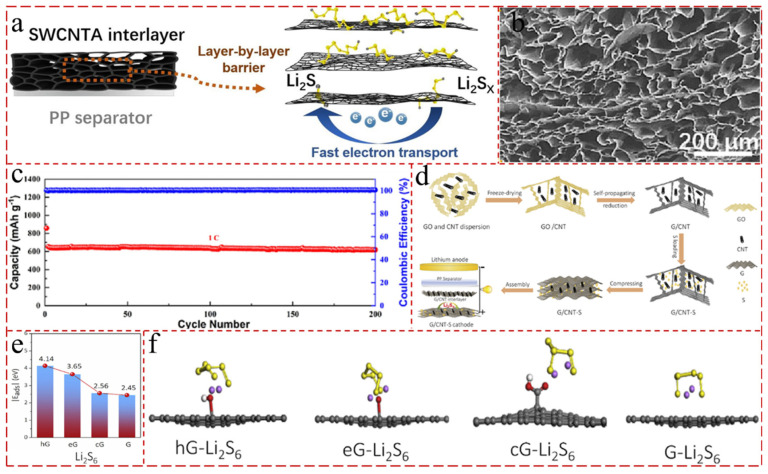
(**a**) Schematic illustration of the physical blocking mechanisms of SWCNTA interlayers [162]. (**b**) SEM images of hierarchical pore structures in SWCNTAs [162]. (**c**) Long-term cycle stability of LSBs with SWCNTA interlayers at 1 C (the first cycle is at 0.2 C) [162]. (**d**) Schematic of the synthesis of G/CNT-S//G/CNT cathode-integrated G/CNT-S host with G/CNT interlayer for Li single-bond S batteries [163]. (**e**) Theoretical absorption energy of Li_2_S_6_ interacting with hG, eG, zG, and graphene (G) [163]. (**f**) Li_2_S_6_ adsorption geometries on hG, eG, cG, and G (carbon, sulfur, oxygen, hydrogen, and lithium atoms are represented by grey, yellow, red, white, and purple, respectively) [163].

Separators, as an indispensable component of batteries, play an important role in obstacle polysulfides at the cathode side and preventing them from shuttling to the anode to react with the lithium metal [158,159,160]. Various separators and functional interlayers have been developed to inhibit the diffusion of polysulfides in LSBs. For instance, the MoS_2_/CNT interlayer provided the LSBs with excellent conductivity and efficient barrier for the LiPSs, and displayed a high initial capacity of 1237 mA h g^−1^ and a ultra-low capacity decay of 0.061% per cycle over 500 cycles at 0.2 C [161]. Given the excellent performance of the CNT interlayer in LSBs, various CNT aerogels are adopted as interlayer in LSBs. Li et al. [162] fabricated hierarchical lamellar SWCNT aerogels by freeze-casting as multifunctional interlayers to capture polysulfides and protect anodes in LSBs. Due to their unique physical structure and large specific surface, SWCNT aerogels can successfully suppress the shuttling effect of LiPSs by layer-by-layer blocking (Figure 7a–c). And the LSBs with SWCNTA interlayers maintained a high specific capacity of 559 mAh g^−1^ at a sulfur loading of 10.35 mg cm^−2^ after 60 cycles. Another important work was reported by Shi et al. [163]. They cleverly synthesized compressed graphene/CNT (G/CNT) aerogels to serve as free-standing, compact, conductive, and integrated cathodes and simultaneously as the interlayer for LSBs. The G/CNT aerogels displayed a 3 D interconnected porous network, large surface area (363 m^2^ g^−1^), high electrical conductivity (67 S m^−1^), and robust adsorption for LiPSs, which provided the LSBs with a high initial capacity of 1286 mAh g^−1^ at 0.2 C and low decay rate of 0.06% over 500 cycles at 2.0 C (Figure 7d–f). Due to strong electrical conductivity, admirable mechanical properties, customizable surface chemistry, excellent charge storage capacity, and prompt ion diffusion kinetics, MXene is widely used in the energy storage fields, comprising lithium-ion, sodium-ion, and potassium-ion cells and LSBs, etc. [164,165,166,167]. Yin et al. [168] chose CNT/MXene aerogel to modify a separator for LSB, where MXene was as the active matrix and CNTs acted as the conductive pillars to immobilize and promote LiPSs. The LSBs assembled with CNT/MXene aerogel separators delivered a high rate capacity of 1043.2 mAh g^−1^ at 2 C and an excellent cycling life of over 800 cycles at 0.5 C with a low capacity decay rate of 0.07% per cycle.

## 5. Graphene Aerogels

### 5.1. Synthesis and Characterization of GAs

**Figure 8:**
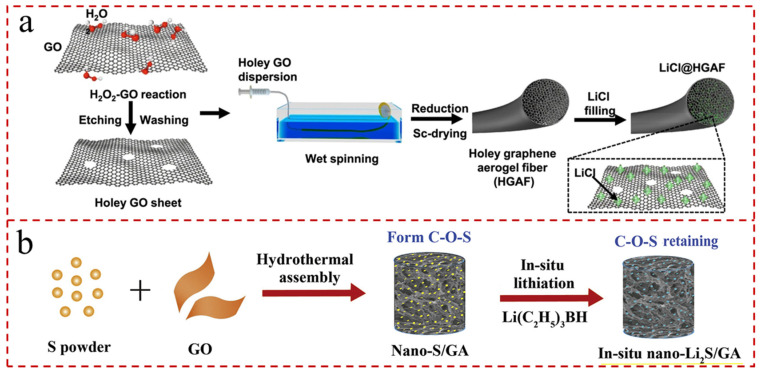
(**a**) Schematic illustration of the fabrication of hygroscopic holey graphene aerogel fibers, which were obtained by wet spinning, HI reduction, and supercritical drying. LiCl was introduced by simple impregnation [176]. (**b**) Schematic illustration of the fabrication of Li_2_S/GA composite electrodes by in situ and ex situ processes [178].

Due to properties such as excellent electrical conductivity, ultra-high Young’s modulus, good flexibility, high theoretical surface area (2630 m^2^g^−1^), and porosity, graphene is one of the most promising candidates for the preparation of cathodes for LSBs [169,170]. With the advancement of science and technology, graphene has become available in various forms, including layered graphene (LG), GO, reduced GO (r-GO), and graphene nanoribbons, etc. [171,172,173,174]. The graphene can accommodate large amounts of sulfur and inhibit the shuttling effect of LiPSs due to physical and chemical effects. However, graphene with a 2 D structure is prone to agglomeration, which can reduce the dielectric constant and increase interfacial polarization and π-electron stacking on the substrate surface, thereby further leading to unfavorable electron transfer and decreased electroactivity [175]. In order to address this issue, various graphene examples with 3 D structures have been cleverly designed and explored. Graphene aerogel (GA), with a typical 3 D structure, consists of graphene sheets with a porosity of up to 99.8%. GAs integrate the inherent excellent properties of discrete graphene sheets with the unique properties of porous materials, including conspicuous specific surface area, ultra-light weight, hierarchical microporous structure, intriguing mechanical properties, and electrical conductivity, etc. For example, Hou et al. [176] described hygroscopic holey GA fibers with integrated functionalities of excellent mechanical performance and superior specific surface area (Figure 8a). And Yin et al. [177] fabricated a polyimide fiber-reinforced GA (PINF/GA) with a 3 D interconnected structure via one-pot compounding and in situ welding. And PINF/GA possessed intensive structural stability under a large strain compression (99%) and thermal-conductivity change ratio as high as 9.8.

### 5.2. Pure GAs

Due to the prominently conductive charge transfer network with flexible and porous structure, the 3 D structure of GAs is considered to as an efficient sulfur host to accommodate the volume expansion of LSBs during the charging and discharging process. Inspired by this, Wang et al. [178] synthesized GA loaded with sulfur as a cathode by hydrothermal method and the obtained S/GA nanoparticles displayed a large specific surface area, uniform dispersion, and robust interaction between S nanoparticles and GA (Figure 8b). The battery assembled with the S/GA nanoparticles exhibited a high specific capacity of 838.5 mAh g^−1^ at 0.1 C. Currently, it is indispensable to flexible, thin, and lightweight cathodes for LSBs. In general, binders used in conventional cathodes can reduce the conductivity and lower the specific capacity of the LSBs. Therefore, the construction of free-standing cathodes without binders and collectors has aroused great interest. For example, Carmen et al. [179] described the free-standing r-GO aerogel as a supporting cathode for LSBs, which have a high areal capacitance (up to 3.4 mAh cm^−2^) and delivered initial areal capacity values of 3.25 mAh cm^−2^ and 2.8 mAh cm^−2^ after 350 cycles at a current rate of 0.1 C.

### 5.3. Heteroatom-Doped GAs

**Figure 9:**
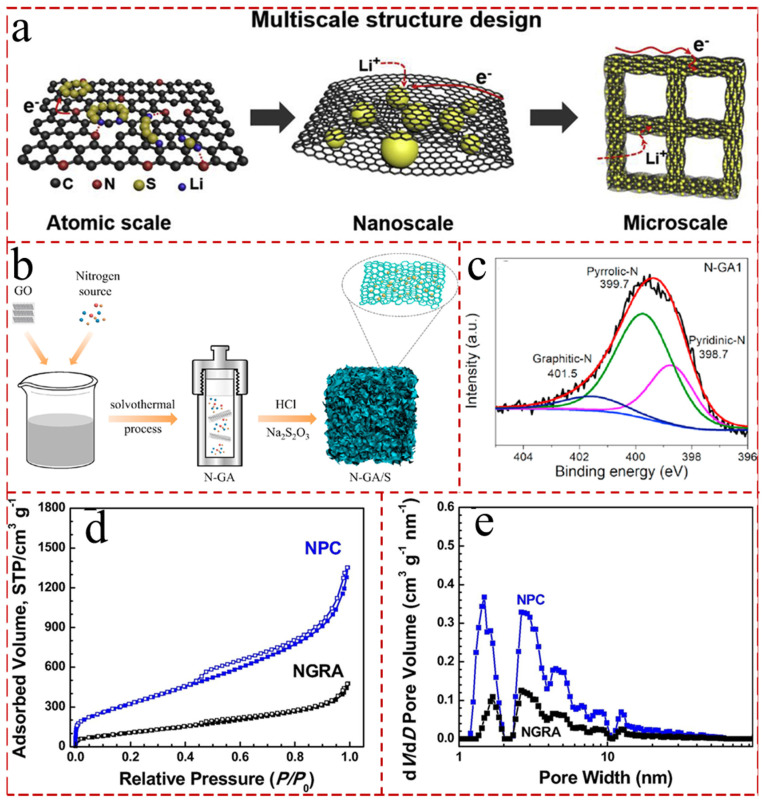
(**a**) Schematic illustration of the multiscale structure design of 3 D PNG as a sulfur host material. (A color version of this figure can be viewed online.) [190]. (**b**) Schematic illustration of the synthetic route for N-GA/S [191]. (**c**) The N 1s XPS spectrum of the N-GA1 [191]. (**d**) Nitrogen adsorption–desorption isotherms of NGRA and NPC [192]. (**e**) Pore size distribution profiles calculated by the NLDFT method of NGRA and NPC [192].

Although graphene aerogel shines in LSBs, the weak interaction force between with non-polar graphene and polar LiPSs cannot effectively capture and immobilize LiPSs. Therefore, doping graphite aerogels with non-polar non-metallic atoms is an effective means to capture LiPSs and reduce the loss of active substances [180,181]. In general, the heteroatoms ought to possess the following elements: strong non-polarity, strong electronegativity and small atomic radius, etc. [182,183]. The main non-metallic doping atoms commonly include: nitrogen [184], boron [185], sulfur [186], fluorine [187], phosphorus [188], and chlorine [189]. Among them, nitrogen (N) atom doping is the most commonly used method for graphene aerogels to inhibit the LiPS shuttle effect due to their abundance of extra electron pairs. Chen et al. [190] described a facile strategy to devise 3 D hierarchical porous nitrogen-doped graphene as a sulfur host material for LSBs (Figure 9a). The N-doped graphene aerogels (N-GAs) have 3 D interconnected hierarchical porosity, conductive networks, and robust mechanical structures, which lead to multi-scale electron/ion transport, sulfur accommodation, and polysulfide confinement. The composite aerogels, as cathodes, exhibited an initial specific capacity of 1311 mA h g^−1^ at 0.2 C, outstanding rate capability (762 at 2.0 C and 580 mA h g^−1^ and 3.0 C), and favorable cycling stability (714 mA h g^−1^ at 1.5 mA cm^−2^ after 400 cycles). Furthermore, Jia et al. [191] designed N-GAs with three different nitrogen sources (urea, ethylenediamine, and ammonia) (Figure 9b). Among them, the N-GAs derived from ethylenediamine had an excellent discharge specific capacity of 723.9 mAh g^−1^ after 100 cycles at 0.7 C and the capacity retention rate was up to 87.4%, while the coulombic efficiency still remained 98% due to the highest proportion of pyridinic-N (Figure 9c). And, the nano-sulfur can increase the contact area with a carbon-based matrix and electrolyte, which significantly improved charge transport, lithium ion diffusion, and the utilization and rate performance. A graphene-coated sulfur layer can inhibit the shuttle effect, enhance battery stability, and increase sulfur loading. Interestingly, Zhou et al. [192] demonstrated the opposite approach of using N-doped graphene nanoribbon aerogel (NGRA) as a source for the preparation of nanostructured porous carbon (NPC). The high Brunauer–Emmett–Taylor specific surface area (1380 m^2^ g^−1^) and the layered porous structure of NPC bestowed LSBs superior electrochemical performance (Figure 9d,e). Although the electrochemical performance of the graphene aerogel can be improved by heteroatom doping, due to the insufficient doping amount (usually less than 10%), the severe shuttling phenomenon of LiPSs cannot be effectively inhibited. Therefore, the doped aerogels are generally compounded with other functional materials to further improve electrochemical properties, which is discussed in detail below.

### 5.4. Composite GAs

And the pure GAs have the disadvantage of poor mechanical properties and a skeleton that is prone to collapse. In contrast, untreated pure GAs exhibit highly brittle and friable mechanical properties due to their ultra-high porosity, the skeleton is prone to collapse, and most aerogels are hydrophilic due to the presence of hydroxyl groups on the surface and are prone to water absorption [193,194]. GAs could not effectively inhibit the “shuttle effect” of soluble LiPSs nor ameliorate the cathodic passivation phenomenon and catalytic slow reaction kinetics due to the precipitation of the reduction product Li_2_S [195,196]. In order to improve these defects, inorganic materials (e.g., transition metal oxides (TMOs), transition metal sulfides (TMSs), bimetallic compound have been added uniformly to aerogels during preparation to achieve optimal properties. For example, He et al. [197] introduced a double template method that integrates both ice and emulsion templates to acquire GAs with distinct orderly hierarchical structures, and subsequently deposited MXene onto the GA surface to prepare MXene/graphene composite aerogels (MGAs). The MGAs displayed higher strength than GA-x due to MXene’s effective improvement of the incomplete pore structure.

#### 5.4.1. GA with TMOs

Due to their inherent oxygen-rich surface structure, transition metal oxides (TMOs) have a strong affinity to LiPSs. However, TMOs have an insufficient surface area to host a large sulfur amount, low conductivity, and poor electrochemical performances. In order to solve the above problems, decorating TMOs in 3 D highly porous carbonaceous materials is one of the most promising tactics. Among them, in view of the high surface area and excellent electrical conductivity, graphene aerogels can act as porous matrixes for sulfur immobilization [198,199]. In addition, TMOs are responsible for providing additional sites for chemical interaction with LiPSs [200,201].

**Figure 10:**
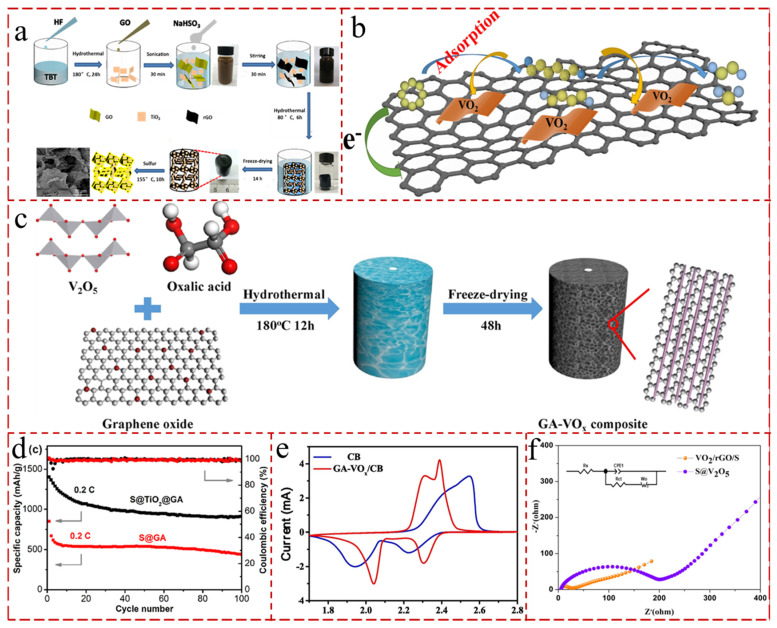
(**a**) Schematic illustration of the preparation process of S@TiO_2_@GA [203]. (**b**) Schematic illustration of the preparation of the GA-VO_x_ composite [206]. (**c**) Schematic illustration of the mechanism of VO_2_/rGO/S exhibiting both LiPS adsorption and good charge transfer capability [207]. (**d**) Cyclic performance of S@TiO_2_@GA and S@GA electrode at 0.2 C during 100 cycles [203]. (**e**) CV curves [206]. (**f**) Electrochemical impedance spectroscopy of the VO_2_/rGO/S and S@V_2_O_5_ electrodes [207].

Titanium dioxide (TiO_2_) is considered to be one of the most promising metal oxides to exacerbate the chemical wrap of intermediate LiPSs and to accelerate redox reaction kinetics [202]. Wang et al. [203] described a 3 D porous graphene aerogel decorated with a high exposure of anatase TiO_2_ (001) nanoplatelets as the cathodes for LSBs (Figure 10a). Compared with the conventional TiO_2_ (101) nanoparticles, the lattices of TiO_2_ (001) nanosheets and graphene (002) nanosheets are highly matched to easily produce sufficient heterojunction interfaces, which can expedite the fast electron transport in the interfaces. The fabricated S@TiO_2_@GA cathode had an excellent initial discharge capacity of 1404 mAh g^−1^ and possessed 905 mAh g^−1^ after 100 cycles at 0.2 C, which is rather higher than S/GA composite (810 mAh g^−1^ and 495 mAh g^−1^ after 100 cycles) (Figure 10d). Shaymaa et al. [204] ingeniously engineered N-GAs interconnected with defect-rich narrow TiO_2_ nanotubes as sulfur hosts; the TiO_2_@N-GAs cathode with oxygen vacancies can anchor LiPSs via a forceful chemical interaction, enhance catalytic performance, and accelerate LiPS transformation. The cathode manifested a notable primary specific capacity of 1370.2 mAh g^−1^ at 0.2 C.

Vanadium oxide (V_2_O_5_) is also considered to be a candidate with conspicuous affinity for LPSs, which can extend the lifetime of LSBs. V_2_O_5_ has brilliant electrocatalyst catalytic effects on the adsorption–transformation–diffusion process of LiPSs, while VN can significantly bind to LiPSs with rapid electron transfer and high electrocatalytic activity [205]. Zhang et al. [206] embedded VO*_x_* nanorods in GA (GA-VO_x_) as dual-functional separators in LSBs (Figure 10b). The embedded VO*_x_* can not only chemisorb LiPSs, but also catalyze LiPSs into short-chain Li_2_S. The LSBs assembled with GA-VO_x_-modified separators exhibited distinct cycling performance (708 mAh g^−1^ after 200 cycles at 0.2 C) (Figure 10e). And Chen et al. [207] designed a VO_2_/rGO aerogel as a cathode matrix for LSBs, and the assembled battery still provided a stable specific capacity of 493.4 mAhg^−1^ at 0.5 C after 700 cycles (Figure 10c,f).

#### 5.4.2. GA with TMSs

**Figure 11:**
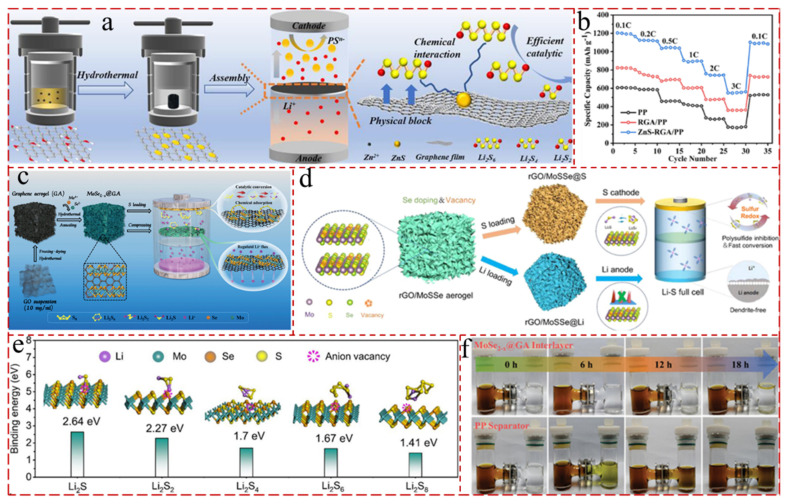
(**a**) Schematic illustration of the synthesis process of ZnS-RGA composite and its synergistic effect of efficient PS blockage and PS conversion catalysis in the LSB [214]. (**b**) Rate performance of the cells with PP, RGA/PP, and ZnS-RGA/PP separators [214]. (**c**) The preparation and mechanism diagram of the MoSe_2−x_@GA/S interlayer integrated electrode [38]. (**d**) Schematic of the sulfur- and Li-loading processes in a vacancy-rich rGO/MoSSe aerogel for the cathode and anode of LSBs [220]. (**e**) Optimized geometries for Li*_x_*S*_n_* species on MoSSe(V) [220]. (**f**) H-bottle visualization shuttle test [38].

In recent years, transition metal sulfides (TMSs) have attracted the interest of researchers for their ability to improve the electrochemical performance of LSBs. Like TMOs, TMSs can promote conspicuous interactions with polar LiPSs and establish sufficiently reactive sites for their redox reactions during cycling due to the conductive substrates and the rapid charge transfer in their structures [208,209]. Nevertheless, similar to TMOs, the majority of pristine TMSs manifest low conductivity, which adversely affects reversible chemical transformations processes for captured Li_2_S_x_ and ultimately brings about decreased sulfur utilization [210,211,212,213]. Liu et al. [214] developed a ZnS quantum dot/reduced graphene aerogel (ZnS-RGA) modified separator for LSBs. ZnS can chemically bond with LiPSs and catalytic sites, while the 3 D porous RGA further physically blocks the migration of LiPSs (Figure 11a). And, the LSBs with a ZnS-RGA-modified separator revealed a high initial discharge capacity of 1211 mAh g^−1^ at 0.1 C and stable cycling performance over 500 cycles at 1.0 C (Figure 11b).

Due to its large specific surface area, adsorption, and ability to accelerate Li_2_S_n_ conversion, 2 D-layered transition MoS_2_ is one of the most promising candidates as a multifunctional electrocatalyst for LSBs [215,216]. In order to overcome the intrinsic defects of aggregation and low conductivity, Hou et al. [217] fabricated RGO aerogels decorated with MoS_2_ nanoporous spheres with abundant active edges exposed as a sulfur host. The S@MoS_2_@GA-0.425 cathode had an initial capacity of 688 mAh g^−1^ and retained a high discharge capacity of 565 mAh g^−1^ after 200 cycles at 0.2 C. In addition, homogeneously distributed other layered TMSs like, CoS_2_, WS_2_, and VS_2_ with GAs act as reactive sites for the chemical bonding of Li_2_S_x_ to reduce the loss of active material and provide convenient diffusion paths for Li^+^.

Due to their good chemical stability and unique structure, as well as their ease of fabrication, MoSe_2_, similar to MoS_2_, is widely used in LSBs. Nevertheless, the substrate of MoSe_2_ has ordinary lithophilic and sulfophilic properties, leading to the unsatisfactory catalytic activity and deposition of lithium [218,219]. In order to address this dilemma, doping heteroatoms and defect engineering are adopted to enhance lithophilicity and sulfophilicity. Li et al. [220] designed a multifunctional host with vacancy-rich MoSSe vertically grown on reduced graphene oxide (MoSSe/rGO) aerogels both as cathode material and anode protection layer (Figure 11d). The embedding of Se into a MoS_2_ lattice was introduced to improve the inherent conductivity and to generate abundant anion vacancies to endow the 3 D conductive GAs with specific sulfiphilicity–lithiophilicity. And the assembled LSBs based on MoSSe/rGO aerogels had a high sulfur utilization and long cycling stability over 1000 cycles and attained a high energy density under more practical conditions (E/S, 4.8 μL mg^–1^; S, 6.5 mg cm^−2^) (Figure 11e). Zhai et al. [38] also engineered Se vacancy-rich molybdenum selenide-modified GAs (MoSe_2−x_@GAs) to serve as both freestanding interlayers and cathode host for LSBs (Figure 11c). Consequently, the defect-rich MoSe_2−x_ with sulfiphilic–lithiophilic properties accelerated the nucleation and dissociation of Li_2_S, while the inserted bifunctional interlayer not only promoted the adsorption and transformation of polysulfides, but also moderated homogeneous lithium deposition and inhibited the growth of lithium dendrites (Figure 11f). The assembled LSBs with MoSe_2−x_@GAs@S electrode and MoSe_2−x_@GAs interlayer possessed a high initial discharge capacity of 1256.9 mA h g^−1^ at 0.2 C and a slow decay ratio of 0.024% per cycle at 1 C after 1000 cycles.

#### 5.4.3. GA with Bimetallic Compound

**Figure 12:**
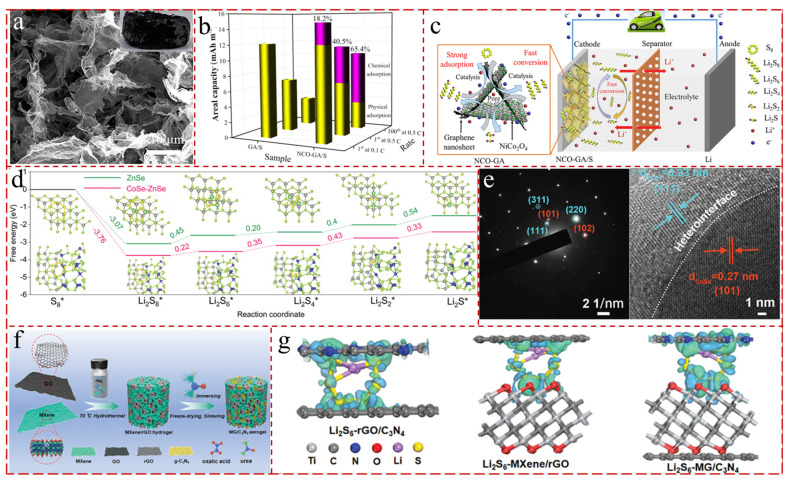
(**a**) The SEM images for NCO-GA [223]. (**b**) The areal capacities of GA/S and NCO-GA/S cathodes at various rates [223]. (**c**) Electrochemical performance enhancement mechanism of NCO-GA composite for NCO-GA/S cathode in the lithium sulfur battery [223]. (**d**) Relative free energy for the reduction S_8_ to Li_2_S on the ZnSe and CoSe-ZnSe heterointerface (insets: the optimized adsorption structures of sulfur species on the ZnSe and CoSe-ZnSe substrate) [224]. (**e**) SAED diffraction pattern and HRTEM image of CoSe-ZnSe [224]. (**f**) Schematic diagram of the synthesis of the MG/C_3_N_4_ aerogel [96]. (**g**) Electron density difference plots of the Li_2_S_6_ adsorbed on rGO/C_3_N_4_, MXene/rGO, and MG/C_3_N_4_ [96].

Beyond these, there are a number of bifunctional bimetallic metal compounds that both adsorb LiPSs and catalyze the conversion of LiPSs, and are also used in LSBs. Compared to conventional binary TMOs, the ternary spinel compound NiCo_2_O_4_ exhibits a better electrochemical activity and electrical conductivity, which stems from the coexistence of the two cations in the crystal structure, and the polarity and catalytic activity of NiCo_2_O_4_ can favor the adsorption of the polar intermediate LiPSs and its subsequent catalytic conversion [221,222]. For example, Tian et al. [223] proposed monodisperse polar NiCo_2_O_4_ nanoparticles decorated the porous graphene aerogel composite (NCO-GA) as sulfur host, which confirms high conductivity, hierarchical porous structure, distinguished chemisorption capacity, and excellent electrocatalytic ability, thereby effectively inhibiting the “shuttle effect”, facilitating ion/electron transport and exacerbating the reaction kinetics (Figure 12a). The NCO-GA/S cathode displayed high discharge specific capacity (1214.1 mAh g^−1^ at 0.1 C), brilliant rate capability (435.7 mAh g^−1^ at 5 C), and conspicuous cycle stability (decay of 0.031%/cycle over 1000 cycles) (Figure 12b,c).

The heterojunctions, a construction of heterogeneously coupled nanocrystals with different bandgaps, have been widely used in LSBs, which is attributed to its ability to provide the comprehensive improvement of the redox reactivity in terms of factors such as LiPSs adsorption, ion diffusion, and electron transfer in LSBs. Ye et al. [224] proposed graphene aerogels decorated with CoSe-ZnSe heterojunction as cathodes in LSBs, which can catalytically accelerate bidirectional sulfur conversion reactions, effectively immobilize sulfur species, facilitate Li ion diffusion, and lower the energy barriers of sulfur reduction and Li_2_S oxidation (Figure 12d,e). And the CoSe-ZnSe@G/S cathode had high areal capacities, good rate capability, and excellent cycle stability with a capacity decay rate of 0.027% per cycle over 1700 cycles.

In summary, graphene aerogels can be compounded with a large choice of metal-containing additives to form composite aerogels to improve the electrochemical performance and cycling stability. Among them, TMOs and TMSs are most widely used to accommodate sulfur and modify separators, which is ascribed to the additional abundant sites for lithium ion anchoring and stronger adsorption energy for lithium ions. However, these materials are characterized by poor ionic/electronic conductivity, which leads to a low sulfur utilization and coulombic efficiency (Table 1). Transition metal nitrides (TMNs) and phosphides (TMPs) have better electrical conductivity, electrocatalytic properties, and intrinsic ability to chemically trap LiPSs through the construction of specific bonds (V-N, S-Ti-N, P-O, etc.), which is also favorable to the development of LSBs.

#### 5.4.4. GAs with Multi-Components

MXene, as a new type of 2 D transition metal carbide/nitride, possesses the features of metal conductivity, structural diversity, and abundant terminal groups [225]. Its high conductivity facilitates rapid electron conduction and accelerates the kinetic process, thus improving the rate performance; the multiple morphology avoids stacking among 2 D MXene nanosheets, which can provide more adsorption/catalytic sites; and the abundance of functional groups facilitates polysulfide anchoring and catalysis in LSBs. Yang et al. [226] constructed the conductive composite aerogels as the cathodes for LSBs by combining GO nanosheets and MXene nanosheets. The GO/MXene(GM) aerogels possessed adequate holes formed by 3 D interoperable conductive network, which can facilitate rapid lithium ion diffusion and electron transfer. The prepared GM electrode successfully experienced 8 months (almost nine months) at 0.1 C, provided a high initial capacity of 1255.62 mAh·g^−1^, and maintained 615.7 mAh·g^−1^ after 450 cycles. Additionally, Yang et al. [96] developed a 3 D composite aerogel by integrating MXene, rGO, and g-C_3_N_4_ as cathodes (Figure 12f). Benefiting from the unique properties of each component, the MXene/rGO/C_3_N_4_ composite featured excellent mechanical flexibility, high electrical conductivity, enhanced charge–transfer capabilities, and strong interaction with LiPSs (Figure 12g). The Li_2_S_6_@MXene/rGO/C_3_N_4_ cathodes exhibited a high capacity of 1364 mAh g^−1^ at 0.2 C with a capacity retention of 1283 mAh g^−1^ after 100 cycles and a rate performance of 1167 mAh g^−1^ at 2 C.

## 6. Summary and Future Perspectives

In conclusion, this review explores carbon-based aerogels in LSBs. Firstly, the preparation of carbon-based aerogels is discussed. Then, the synthesis strategy and the use as LSBs cathodes and interlayers of carbon nanofiber and CNTs aerogels are outlined and analyzed. Finally, for optimization and improvement, two major aspects of heteroatom-doped GAs and composite GAs (TMOs, TMSs, bimetallic compound, and multi-components, etc.) are combined with the excellent properties of graphene-based aerogels. Despite LSBs having had fascinating promise in recent years, carbon-based aerogels as sulfur hosts, cathodes, separators, and interlayers for LSBs are still in the initial stages of LSB development, and there are a number of obstacles yet to be tackled. And there are still several issues need to be solved for their further advancement:

(1) Compared to GAs and CNT aerogels, carbon nanofiber aerogels are underdeveloped, which is mainly related to the manufacture technology of carbon nanofibers. Therefore, the development of carbon nanofibres, particularly derived from polymer nanofibers, is highly important. For example, the development of novel spinning needles to increase the yield of polymer nanofibers.
(2) The generally low mechanical properties of aerogels restrict their use in complex environments; so, a comprehensive strategy is needed to enhance their mechanical properties, from molecular design to macroscopic structure. At the molecular level, introducing flexible siloxane segments or constructing a dual-network, cross-linked structure can enhance the material’s inherent toughness effectively. At the mesoscale, a biomimetic hierarchical structure design can optimise stress distribution. At the macroscale, composite designs incorporating carbon fibre frameworks can withstand extreme loads.
(3) The manufacturing process of most carbon-based aerogels is complex and costly, which makes it difficult to apply in practice, especially in some underpriviledged and distant regions. The development of most aerogel materials has been only limited to the laboratory, and many aerogel devices are too miniaturized for large-scale application. Therefore, aerogels will move towards the direction of being applied in large areas, low cost, and emerging technologies.
(4) Carbon-based aerogels are predominantly employed as sulfur hosts, functional separators, and freestanding interlayers in LSBs. And the utilization of carbon-based aerogels in anodes is still in its infancy. However, it is essential that the meticulous carbon-based aerogel design is adopted to guarantee the decent protection of lithium anodes under high sulphur and current density conditions. One of the main problems with lithium metal anodes is electrolyte consumption and anode corrosion caused by a high E/S ratio. Constructing a thin film of aerogel-based solid electrolyte on the anode is a viable solution to reduce side reactions.
(5) Solid-state LSBs are currently undergoing in a a boom in development to address the severe shuttle effect of LSBs. The low-density solid polymer electrolytes possess high gravimetric energy density, considerable design flexibility, and outstanding contact with electrodes and have attracted extensive attention. One major ambitious goal is the integration of aerogel materials as polymer electrolytes into LSBs. It is indispensable to establish the high ionic conductivity, excellent interfacial compatibility, and wide electrochemical window of the ideal aerogel-based electrolyte with both sulfur cathode and lithium anodes. Therefore, developing aerogel composite electrolytes has become a promising future development.
(6) Many laboratory-scale studies of LSBs are limited to coin cells and lack the performance associated with practical multilayer pouch cell configurations. And few studies demonstrated promising the performance of aerogels in high sulfur loading at the pouch cell level, and there is a lack of data on multilayer pouch cells. Therefore, it is critical to scale up successful aerogel optic technology for high loaded lsbs from the coin cell level to adequately address the challenges and requirements of multilayer pouch cells for commercial prototyping.
(7) Advanced characterization techniques, such as XRD, Raman spectroscopy, TEM, and in situ XAS, etc., should be employed to monitor and visualize the transformation of intermediates and the concentration of soluble polysulphides at different stages of the LiPS conversion process in real time, providing direct data to gain a deeper understanding of the electrochemical reaction pathways. Meanwhile, machine learning can accelerate the discovery of functional carbon-based aerogel materials by recommending experimental conditions that quickly achieve the target performance when screening adsorbents and catalysts.

## Figures and Tables

**Table 1:** The electrochemical characteristics of high areal sulfurloading cathodes with modified carbon-based aerogels.

Electrodes	Sulfur Loading (%/mg cm^−2^)	Initial Capacity (mAh g^−1^)	Cycling Number	Final Capacity (mAh g^−1^)	Ref.
FeSA-NC@CBC	81.2/5	1006.2 (1 C)	840 (2 C)	799.8 with 79.5% retention after 500 cycles at 0.05 C	[127]
Pt/S/OCNF	77.5/-	1188.1 (0.5 C)	813.2 (2 C)	982.3 with 82.6% retention after 200 cycles at 0.5 C	[50]
S@WCNTAs	63.33/8.02	1018 (0.5 C)	609 (1 C)	a capacity of 559 after 60 cycles at 0.1 C	[95]
CNT@Li_2_S_8_	43/5	1388.2 (0.1 C)	1268.9 (2 C)	899.9 with 64.8% retention after 60 cycles at 1 C	[78]
MXene/CNT/MXene	-/7	712 (0.5 C)	-	570 with 80% retention after 800 cycles at 0.5 C	[110]
3D CNT/Ti_3_C_2_T_x_	70/-	1329.7 (0.5 C)	1043.2 (2 C)	a capacity retention of 64% after 800 cycles at 0.5 C	[111]
NFC/CNT	83.3/2.3	1143 (0.1 C)	1675 (1 C)	704.9 with 85% retention after 100 cycles at 1 C	[112]
N-GA/S	75.5/-	1210.7 (0.1 C)	610 (3 C)	724 with 89% retention after 100 cycles at 0.7 C	[138]
FeP/rGO/CNTs -S	75/3.5	1271.6 (0.1 C)	613.1 (3 C)	1038.4 with 81% retention after 100 cycles at 0.1 C	[133]
FeP/rGO/CNTs	60/9.6	1312.3 (0.1)	647 (2 C)	-	[141]
S@TiO_2_@GA	55.2/-	1404 (0.2 C)	-	-	[203]
GA-VOx	80/2.6	1057 (0.05 C)	442 (2 C)	734 with 69% retention after 140 cycles at 0.2 C	[152]
ZnS-RGA/PP	66/3.1	1211 (0.1 C)	794 (2 C)	865 with 71.4% retention after 100 cycles at 0.2 C	[157]
NCO-GA/S	80.4/-	1241.1 (0.1 C)	435.7 (5 C)	a capacity retention of 68.5% after 200 cycles at 0.5 C	[163]
CoSe-ZnSe@GA	66.2/7.7	1654 (0.1 C)	808 (3 C)	a capacity retention of 88.8% after 108 cycles at 0.2 C	[166]
GM	53/2	1255.62 (0.2 C)	974.62 (2 C)	615.7 with 51% retention after 450 cycles at 0.1 C	[134]
MG/C3N4	-/4.92	1315.6 (0.2 C)	1167.4 (2 C)	a capacity retention of 97.5% after 100 cycles at 0.2 C	[167]
MoSSe/r-GO aerogel	-/6.5	938.8 (0.5 C)	-	637.3 with 66% retention after 1000 cycles	[172]
MoSe_2−x_@GA/S	-/4.8	1256.9 (0.2 C)	931.7 (2 C)	a capacity retention of 76% after 1000 cycles at 1 C	[173]
S/Co-GC@GPCA	63.33/2.03	939.9 (0.1 C)	439.1 (2 C)	677.3 with 72.1% retention after 300 cycles at 0.1 C	[182]

1 C = 1675 mAh g^−1^.

## Data Availability

No new data were created or analyzed in this study.
